# ‘I should have thought that Wales was a wet part of the world’: Drought, Rural Communities and Public Health, 1870–1914

**DOI:** 10.1093/shm/hkw118

**Published:** 2016-12-21

**Authors:** Keir Waddington

**Keywords:** drought, public health, rural, Wales, water supply

## Abstract

From 1884 onwards, Britain experienced a series of major droughts, which reached their peak in the ‘Long Drought’ (1890–1909). Despite being imagined as a wet part of the world, rural Wales was hard hit as many communities did not have access to reliable water supplies. As medical officers of health and newspapers talked about water famines, alarm focused on questions of purity and disease as drought was presented as a serious health risk. Using rural Wales as a case study, this essay explores vulnerabilities to water scarcity during periods of drought to examine the material and socio-political impact of water scarcity and the resulting public health problems faced in rural areas. In addressing how droughts in rural communities were physical and social phenomena that generated considerable alarm about infectious disease, this essay also reveals how periods of water scarcity were an important determinant in improvements to rural water provision.

In a debate over improvements to the water supply for the village of Henllan in Denbighshire in 1890, Major-General C. Phipps Carey, one of the deputy engineering inspectors for the Local Government Board (LGB), argued that the proposed scheme was excessive. In defending his conclusion, he explained: ‘you don’t have droughts in this part of the country … . I should have thought that Wales was a wet part of the world’.^1^ If Phipps Carey’s assumptions reflected representations of the Welsh landscape found in Romantic paintings and travel writing, the inhabitants of Henllan were clearly worried about the effects of drought on their water supply. Yet Henllan was not an isolated case. Two years later comments by the conscientious Ruthin Rural Sanitary Authority revealed how it too was acutely aware that many rural communities in both low-lying and more mountainous parts of the district were ‘painfully acquainted with drought’ and feared for their water supplies.^2^ For a region which covers some 8,000 damp, hilly western square miles of Britain, Wales was not a typical region of risk for droughts given the high average rainfall. Yet rural Wales suffered periods of intense water scarcity in the late nineteenth and early twentieth centuries that disrupted social normalcy. Although rainfall data suggest that Wales faced comparatively deeper drought conditions than the rest of Britain in only seven of the drought years between 1870 and 1911, the effect of water scarcity on rural Welsh communities was significant.^3^ As the hardworking medical officer of health (MOH) for Gwyrfai Rural Sanitary District in Caernarvonshire explained in 1896, ‘the bitter experience of the past’ made those living in rural Wales ‘very anxious’ about the effects of drought.^4^ In response, like their counterparts in urban areas, the inhabitants of rural parishes and villages asserted their need for secure water supplies. However, underpinning their demands was not consumer politics or new ideas of hygiene but experiences and fears about the dangers of drought.

Notwithstanding calls for medical historians to consider ‘how the weather makes us’, the anxieties expressed in places like Henllan and by the Ruthin Rural Sanitary Authority do not figure in studies of water supplies.^5^ Research has drawn attention to the contested nature of municipal supplies and the relationship between public health and the disciplinary power of cleanliness, highlighting the material dimension of urban water infrastructures and how connecting houses to sanitary networks facilitated cultures of self-regulation, cleanliness, and individualisation. Where questions of water scarcity have been discussed they have invariably been associated with the unequal development of municipal infrastructures, even though the period 1870–1911 saw repeated droughts that threatened local water supplies.^6^ Equally, although there is a substantial literature on drought as a natural disaster in Africa, Asia, and the Americas, the impact of drought in Britain has attracted little attention beyond a handful of studies, many of which have approached drought from a meteorological or hydrological perspective.^7^ As Douglas shows in his environmental history of cities, environmental hazards and responses to them need to be better understood, while we know little about the influence of climatic events on public health in Britain.^8^ This is despite growing scholarship that examines the links between climate variability, social vulnerability, and societal responses, or work on climate history that has advanced debates about how societies have reacted and adapted to weather events.^9^ Yet as Taylor and Trentmann reveal, drought and disruption in the late-Victorian period prompted heated conflicts over water supply, particularly during ‘the Long Drought’ of 1890 to 1909. Drought conditions in Britain often lasted a few months and were not catastrophic events that can be portrayed as manifestations of calamity-sensitive conditions, but they do highlight the gap between technological networks of piped water and everyday practices, revealing how urban water infrastructures were ‘far less durable than imagined’.^10^ Taylor and Trentmann’s work on the East End of London draws attention to the need to consider the role of scarcity in shaping debates about water provision and usage in urban communities, but questions remain about how other communities were affected by drought.

Given that patterns of supply can result in significantly different vulnerabilities to water scarcity, there is a need to look beyond large metropolitan centres to consider the impact of drought on market towns and villages. As *Country Life Illustrated* explained in 1899, drought ensured that rural water supplies were ‘fast becom[ing] the question of the day’.^11^ Rather than drought being primarily an urban phenomenon, this essay shows how rural communities felt the problems of intense or prolonged periods of drought more acutely than their urban counterparts. While scholarship on public health is deeply embedded in the urban, as scholars have started to expose, rural sanitary conditions differed markedly from the bucolic image presented to metropolitan readers. Much of this research has, however, addressed the limits of rural sanitary reform, and far less attention has been directed at water provision or scarcity.^12^ As the case of Henllan suggests, in such communities, water scarcity not only acted as a catalyst for public debate about the nature of rural water supplies, but also provided an important stimulus for extending rural supplies that had little to do with consumer politics.

In shifting attention from the urban to the rural environment this essay offers a new perspective on the material and social impact of drought to reveal what an examination of water scarcity tells us about the role of climatic events in public health. Rural Wales as a region is used here as a case study to uncover both the hardships caused by water scarcity in rural environments and the public health responses to drought to problematise urban narratives surrounding the development of water infrastructures.^13^ To contextualise the nature of rural water provision, the essay begins with an examination of the character of rural water supplies across the region. In revealing the fragility of supplies, it shows how droughts in rural areas were physical and social phenomena to highlight the local and social vulnerabilities to water scarcity and the uncertainties of the rural environment.^14^ The essay then illuminates how, more so than in urban areas, drought in rural communities was explicitly conceived to be a threat to lives and livelihoods as it generated considerable anxiety about outbreaks of infectious disease even if the epidemiological impact was not quantifiable. We still lack detailed work on rural environmental deprivation and the surprisingly high associated levels of infectious disease in rural communities.^15^ An examination of the fears generated by the connections made between it and infectious disease points to the significance of deprivation and disease in rural communities and highlights a different reality from the one presented in contemporary debates about the countryside.^16^ From exploring these connections, the essay finally addresses how drought and the fears it generated provide a crucial context for understanding why, in the last quarter of the nineteenth century, localised sanitary infrastructures to improve water supplies were increasingly put in place in rural communities. At stake here were not piped supplies, consumer politics, or an ‘imaginary drought’, but the interplay of water scarcity, hardship, and localised, low-cost solutions.^17^

## ‘A Wet Part of the World’? Water in Rural Wales

Until the 1830s, many urban communities often relied on surface wells, public pumps and limited piped supplies but the 1840s and 1850s brought increased statutory regulation of water provision and attempts to extend urban supplies. The result was a slow, uneven move towards municipal ownership and the replacement of local sources of supply with piped supplies even if technical barriers, location and class meant that, as Trentmann shows in the *Empire of Things*, running water did not always flow smoothly.^18^ Although investment and technical innovations in urban areas saw new water infrastructures put in place, the same patterns were not replicated in many rural communities. Their inhabitants, especially those living some distance from market towns, mainly took their water from local natural sources rather than from commercial water companies or local authorities. Even with the creation of rural sanitary authorities in 1872, rural sanitary reform remained limited, handicapped by inadequate legislation, limited resources, rural poverty and resistance from landowners.^19^ Investment in rural water supplies was therefore often minimal and many rural communities continued to rely on traditional sources of supply into the Edwardian period. As an investigation into the nature of local government water supplies revealed in 1914, 62 per cent of rural parishes in England and Wales were without piped water.^20^ The result was different vulnerabilities to water scarcity that made rural areas more susceptible to drought as a consequence of local patterns of water supply.

Rather than suggesting a simple narrative of rural backwardness, geology and landscape compounded the limitations of rural sanitary reform in Wales. Many rural sanitary districts incorporated ‘a large area of mountain land’ and with poor transport networks beyond the south Wales coalfield, many contained isolated communities that were hard to reach.^21^ The difficulty and high relative cost of piped supplies for sparsely populated areas made the provision of water commercially unviable for companies and expensive for rural authorities, especially when the immediate benefits were intangible. As a result many communities in rural Welsh had few options other than to use surface wells, springs, streams or stored rainwater. In a paper before the North Wales Sanitarians in 1900, Levi John, sanitary inspector to the Conway Rural District Council in north Wales, explained how the ‘water-supply in Rural Districts is in most cases of a variable character,—some districts are dependent entirely on rainwater stored in wooden tubs and casks, others derive their supply from shallow wells’.^22^ With counties in the region having limited or no underground supplies, and mainly reliant on shallow wells or surface water from upland areas, wells were not always wells in the conventional sense. In the hilly dairy farming district surrounding Aberaeron on the west coast of Wales, for instance, the term ‘well’ was also applied to holes in the banks of streams or roads filled by water percolating into them from the neighbouring topsoil or stream. An inspector for the LGB suspected that ‘spring waters’, which were the main source of supply in the district, came from little more than ‘land of a boggy or water-logged character’.^23^ Nor were sources of water always local: many living in upland and lowland areas had to walk up to two miles to gain access to a well or spring, a distance the LGB felt ‘cannot be regarded as reasonable’.^24^ In the sparsely populated Gower peninsula in south Wales, for instance, people commonly carried water back from nearby wells ‘in utensils to their homes’, while in the village of Cilycwm, Carmarthenshire, the farm workers complained that they had ‘to carry water for dietetic purposes over five fields and have to climb over six stiles’.^25^

Wales may have been widely perceived as a wet and wild place, but as the MOH for the Cardiff Union Rural Sanitary Authority explained in 1886, in most ‘rural districts there is almost always a scarcity of water’.^26^ In Flintshire, the village of Hawarden was reported to be ‘without water for at least 9 months of the year’. The 142 houses in the village had to rely on rainwater, a common solution in response to inadequate supplies, while in the Gower peninsula the water supplies in the 1890s were ‘insufficient to meet the requirements of the summer season’ in a district otherwise troubled by ‘few and trivial nuisances’.^27^ Rural sanitary officials regularly noted how wells in small villages ran dry during an average summer and how it was ‘extremely difficult to obtain water from another source’.^28^ Problems with water supplies were encountered even in large villages, especially during the summer: for instance, those living in Llannon in Cardiganshire depended ‘entirely for their supply’ in summer ‘on the two little streams of fifth that pass through large village’.^29^ It was only towards the end of the nineteenth century that an increasing number of rural communities began to benefit from improved water supplies, and even then provision and access often remained problematic.^30^

Even when rural communities had access to water, the quality of rural supplies was poor. The first half of the nineteenth century had seen concerns about water framed around cultural notions of purity and cleanliness as critics of commercial ventures demanded ‘pure water’. While, as Hamlin explains, considerable uncertainty surrounded what ‘pure’ and ‘clean’ water meant, rural supplies were often problematic in both senses.^31^ If water from isolated and mountainous regions could be imagined as examples of ‘perfect purity’, investigations found that many local wells and springs in rural Wales were ‘not in a satisfactory state’ and often ‘tainted’.^32^ Rural supplies were prone to pollution: many rural wells were shallow and believed to be ‘unquestionably within the area of subsoil pollution’.^33^ Contamination from nearby cesspools, pigsties, farmyards, cows, sheep and other nuisances was common. Reports regularly emphasised how water supplies for villages were as a consequence ‘of a very doubtful character’, as for instance in Cilcennin in mid-west Wales where the village pump, which supplied the 423 people living there, was ‘within a few feet of the churchyard, and is much below some of the burial ground’.^34^ Industrialisation, which intensified after 1880, also had a marked effect on water supplies. Coal mining, as well as metal works in south Wales and slate quarrying in north Wales, had an ecological and environmental impact, scarring the landscape and harming rural water supplies. Ironworks and other industries choked streams with refuse and Welsh rivers were reported to be the most polluted in Britain.^35^ Mining both polluted and disrupted local water supplies. For example, in Carmarthenshire, the springs in the agricultural land surrounding Penygroes were ‘drained by the numerous coalpits that have been sunk in the neighbourhood’ leaving the area in ‘much need of [a] water supply’.^36^ In rural parts of south Wales, such as the Garw valley, the pollution caused by mining and other industries ensured that pure water remained scarce throughout the late nineteenth century.^37^ Under these conditions, sanitary officials were aware that many living in rural communities often had little choice but to ‘habitually consume water which is liable to pollution’.^38^

## The ‘Usual Sources of Supply Have Completely Vanished’

If access to water was problematic in rural communities, water quality often poor and, during the summer months, frequently limited, what happened under drought conditions? The definition of drought is partly contingent on its impact on society and on the economy, with drought conditions arising from meteorological events (a deficiency of rainfall) and hydrologically (as an accumulated shortfall of surface and groundwater). Within this definition, nineteenth-century Britain experienced a number of national and localised water famines. Major droughts occurred in 1826, 1854–1860, 1865, 1884–1885, and 1887–1888, with ‘the Long Drought’ of 1890 to 1909 the result of a series of dry and cold winters and El Niño events, which created a cumulative hydrological deficit. A further ‘abnormally dry’ summer was recorded in Wales in 1911.^39^ Severity during longer periods of drought varied: the 1887 drought was the third most severe since 1820 and particularly affected northern and western Britain, while 1893, 1899, 1902 and 1905 were the most acute years of ‘the Long Drought’.^40^ Urban communities experienced periods of shortages and intermittent supplies, particularly during the droughts of 1887 and 1893–1898.^41^ Rural communities had a different social vulnerability to drought and suffered far more. As one writer explained in the *British Medical Journal* in 1891, ‘The inhabitants of some of our large towns are not unfamiliar with a diminution in their usual water supply consequent upon a period of drought, but it is probably chiefly in the rural districts that the phrase “water famine” is completely justified in its ominous significance’.^42^

Drought conditions occurred at a local and regional level with increasing frequency and intensity from 1868 onwards. While Wales was no more prone to drought than other areas of Britain, with water supplies in Wales generally from surface water and reservoirs in upland areas, short duration droughts caused by a single dry season caused marked problems. Rural communities, with already poor or limited water supplies, proved particularly vulnerable. This vulnerability became clear during the ‘remarkable drought’ of 1887.^43^ The drought severely affected the whole of Britain, with studies suggesting that existing water catchment areas were ‘more severely taxed than in those of forty subsequent years’.^44^ While historic rainfall data is not available for Wales as a region, rainfall for southwest England and south Wales fell by 34.4 per cent in 1887 compared to the previous ten-year average, whereas they fell by 33 per cent for England and Wales.^45^ As the drought deepened, water scarcity in rural Wales became a local, regional and national problem as newspapers’ ‘weather talk’ focused on drought with increasing alarm. Given what Harley refers to as the ‘recency effect’ where dramatic weather events seize the popular imagination more than expected seasonal variability, such newspaper coverage shaped perceptions and fears of drought as weather and examples of water scarcity became national news.^46^ As the *Western Mail* explained, getting water in many rural communities in 1887 became as difficult as it had been in the 1840s and 1850s when little water infrastructure existed.^47^ Although upland areas had a more variable climate, which ensured that not all areas were equally affected, newspapers noted how by late June ‘the smaller rivers and mountain streams are dried up’. With increasing shrillness newspapers warned of the imminent collapse of the water supply and the failure of existing reservoirs to meet local needs.^48^

Although the 1887 drought stood out in the popular imagination for its intensity, creating alarm that the ‘water famine’ would continue as rainfall remained low and rural water supplies were depleted for the next three years, the 1890s saw further droughts and ‘dry seasons’.^49^ During the 1893 drought, which was noted for its intensity and duration in Britain and Europe, the rainfall in Wales fell to one twentieth of its usual level and sanitary officials and newspapers repeatedly warned of ‘an absolute water famine’.^50^ While an investment in new and more reliable water supplies by urban authorities ensured that they were better placed to cope with drought, by April 1893 the early severity of the drought in rural Wales produced conditions that had ‘probably never occurred in the memory of any person now living’.^51^ As the drought deepened, rural communities experienced serious water shortages. Conditions were seen as unprecedented: even upland areas—where rainfall was often higher—faced severe shortages.^52^ Sanitary authorities issued warnings to save water and those wasting water were branded as lacking in ‘social morality’.^53^ Reservoirs ran dry and rural communities found themselves without water. In the normally flood-prone Vale of Clwyd in Denbighshire, for example, farming communities found the ‘usual sources of supply [had] completely vanished’ by June, while in the ‘hilly’ pastoral district covered by the Hay Rural Sanitary Authority, the MOH described how ‘water was scarce during the greater part of the year and the supplies completely failed in some villages’. Elsewhere prayer meetings for rain were held.^54^ Increasing alarm was expressed about the effects on agriculture coming as the drought did ‘on top of a period of profound agricultural depression’.^55^ Although drought conditions prompted widespread cries of distress from the agricultural community nationally, rural Wales was felt to be worse hit than other areas of Britain: arable land baked, crops were badly affected, milk supplies started to fail, farmers described going without water for months, and in Pembrokeshire, cattle were reported to be dying in droves.^56^ Rainfall and thunderstorms became major news items, and in southwest Wales the availability of drinking water continued to be limited into November.^57^

If drought conditions were severe in Wales in 1893, rural sanitary officials made repeated reference to the hardships caused by drought throughout the 1890s and 1900s: 1895–1896, 1898, 1901–1902, 1905 and 1911 proved particularly bad years in Wales, with north Wales and the upland areas experiencing notable droughts in 1902, 1904 and 1905 when rainfall was 10 per cent lower than the average low rainfall for the pervious decade.^58^ In many rural communities in Wales repeated droughts during the 1890s ensured that local supplies nearly gave out, with ‘some of the wells being dry for a considerable period’ as ‘rivers and wells are becoming exhausted’.^59^ Although the failure of village wells during droughts was commonly noted in England and Wales, there were incidences of water shortages in rural Wales that were particularly severe. For example, the MOH for the Brecknock Rural Sanitary Authority recorded ‘exceptional distress’ across the pastoral and partly mountainous district owing to the ‘long continuation of this unusually severe weather’ in 1895. Five years later, during the 1900 drought, the inhabitants of Llysfaen parish on the Caernarvonshire coast faced a ‘very serious’ water shortage as ‘there was not a drop of water for either man or beast to be obtained in the locality’.^60^ Complaints about ‘precarious and insufficient’ supplies in rural communities became increasingly vocal as water in ‘many localities [ran] very short’ during the ‘Long Drought’.^61^ Speaking about houses in the rural part of the Rhondda valley, one local official worried that ‘if the drought continues Heaven only knew where they would be’. Such was the severity of the water shortages that many rural communities echoed the same concerns as they feared water famines.^62^

Drought was feared because it disrupted normal domestic and sanitary practices. Levels of hardship are hard to quantify for rural communities where water usage could be as low as 5 gallons per head daily (compared to the 35 recommended) given that many villages had basic drainage—often little more than gutters—and were only gradually moving from privy pits to pails in the 1910s.^63^ However, newspaper and sanitary officials’ statements about water famine and villages going without water for months hint at the difficulties experienced. Reports of deficient supplies conceals how during droughts those living in rural communities would collect water between 3am and 7am or walk longer distances to access meagre supplies; how drains and gutters were not flushed; how ditches became clogged with liquid refuse; how waste water was utilised for cooking and for washing clothes and floors; and how water from sources known to be polluted was used.^64^

## Public Fears: Drought and Disease

Whereas responses to droughts in the 1870s were largely framed in the context of the impact on agriculture, after the ‘almost drought’ of 1885 different concerns emerged in rural Wales that became a central feature of alarm about rural water scarcity and access to drinking water into the Edwardian period.^65^ The creation of rural sanitary authorities under the 1872 Public Health Act combined with the growth of the provincial Welsh press ensured that rural sanitary reform attracted increasing attention. As rural authorities stepped up their efforts to tackle nuisances, their meetings and annual reports were reported at length and rural sanitary problems were discussed in the press with growing frequency. Drought magnified these debates.

Water became a social and national issue in Wales during times of drought. Just as in London, Welsh newspapers initially debated what ‘normal’ and ‘rational’ water usage meant during drought conditions, and whether trade was being favoured over domestic consumers.^66^ Questions about the legitimacy and scope of domestic usage in urban versus rural areas was framed in the context of urban anxieties about water scarcity and remained a small part of the concerns generated by drought in rural communities. When it came to rural communities it was not the failure of private companies or wasteful consumers that dominated headlines and discussions at a local level but the public health dangers associated with scarcity and water supplies.^67^ Alarm focused both on the hardships caused by drought for rural communities, and importantly on questions of purity and disease as ‘medical men’, in the words of one newspaper, gave ‘expression to their sense of peril to the community should any attack of fever or cholera unfortunately occur during the present climatic conditions’.^68^ Drought was presented as a serious public health risk for rural communities.

Although in comparison to the ‘urban penalty’, rural areas were consistently presented as heathier than towns, intensely localised patterns of mortality counter the impression of a healthy rural environment. Repeated outbreaks of typhoid and high levels of diarrhoea were common in a rural environment that commentators at the end of the nineteenth century compared to ‘sordid city areas’.^69^ Periods of water scarcity were identified as an important contributing factor in outbreaks of infectious disease, such as typhoid, as alarm focused not on cleanliness but on the dangers of drinking water from marginal and polluted supplies. As drought became a regular feature of Welsh summers from 1885 onwards, water in upland and lowland rural communities emerged as a ‘great anxiety to those responsible for the public health’ given the ‘foulness’ of the water courses that many rural inhabitants found themselves forced to rely upon for drinking purposes. Although the impurity of rural supplies had troubled sanitary officials in the 1870s, during drought conditions references to the dangers of rural water supplies reached a new intensity. For example, as a result of the 1873–74 drought it was felt that the ‘water became most objectionable’ in the wells supplying the rapidly expanding village of Cogan in south Wales. Twenty years later the residents in the straggling village of Dyffryn at the foot of the Rhinogydd mountain range complained about how ‘they had suffered considerably from Drought last summer’ as they struggled to find ‘water fit to drink’.^70^ As the *Western Mail* explained, by 1893 ‘there is many a local authority fairly at its wits end to provide wholesome water’.^71^ Severe droughts in the past had been accompanied by outbreaks of waterborne diseases, and as fears intensified about water quality as periods of water scarcity became more frequent in lowland and upland areas after 1885, the spectre of outbreaks of infectious disease fuelled alarm about the impact of drought on rural communities.^72^

Newspapers provided an important conduit for voicing alarm about infectious disease and drought, acting as a source of rumour and fear. Writing during the 1887 drought, the *Western Mail* warned readers in June of the ‘imminent risk of disease and especially from infectious disorders’.^73^ In a later edition, the paper explained how ‘nothing since the cholera scare has produced so much anxiety’ as the continued drought.^74^ The association between cholera and drought was to prove a potent and enduring one, especially as responses to earlier cholera epidemics had focused on the need for an abundant water supply to ‘wash away’ the disease.^75^ As cholera swept across Europe six years later, and against the background of the LGB encouraging sanitary authorities to put in place cholera precautions, fears that the disease would quickly find a hold in rural Wales given drought conditions were expressed with growing intensity.^76^ Framed within a strictly sanitary framework rather than around notions of germs, rural sanitary officials and Welsh newspaper reports worried that drought would inevitably lead to outbreaks of infectious disease and cholera. William Williams in *A Sanitary Survey of Glamorganshire* commented upon fears about outbreaks of cholera in rural communities ‘should this weather continue for a few weeks longer’ as normal sanitary practices broke down during drought.^77^ In Swansea Union Rural Sanitary Authority, for instance, the prospect of a ‘hot summer and a possible outbreak of cholera’ made the authority anxious early in 1893 ‘to lose no more time in securing the benefit of a good water supply’.^78^ Faced with water scarcity, the return of cholera in rural communities was openly talked about during the 1893 drought when cholera was epidemic in Europe.^79^

Local epidemics of other infectious diseases in England and Wales were linked directly to drought conditions.^80^ Writing about rural south Wales during the 1899 drought, the *Cardiff Times* explained how ‘so long as there is a scarcity of water there is always the further danger of an outbreak of a pestilential disease through incaution in taking impure and contaminated water for domestic purposes’.^81^ Trafford Mitchell, medical officer for Llangyfelach Rural District Council, had already made similar connections when faced with outbreaks of typhoid in 1896. He believed that the increase in typhoid cases in his district was the ‘direct result of the severe drought’. Typhoid was an insidious, endemic disease in the Victorian period with sporadic epidemic outbreaks. However, given its close association with filth and water, an increase in typhoid cases in rural communities, such as in Rhostryfan in northwest Wales in 1893, was widely attributed to drought conditions.^82^ If typhoid became the major fear for rural sanitary officials during droughts, an increase in cases of diphtheria and ptomaine poisoning were equally blamed on drought. Some intestinal infections, such as salmonella, rose quickly in periods of hot weather as meats decayed faster and the number of flies increased. Local sanitary officials were hence advised to step-up their efforts to inspect ‘shambles, shops and markets’ during droughts.^83^ For rural sanitary officials, drought and disease went hand in hand.

Many rural communities and newspapers accepted this link between drought and an increase in disease. Consequently, as the *North Wales Chronicle* explained, they ‘greatly feared the outbreak of a serious epidemic’ during droughts.^84^ Ratepayers in the small village of Groeslon in Caernarvonshire, for example, were ‘greatly excited’ during the 1893 drought as they ‘feared that an outbreak of fever would take place’. Readers of the *Merthyr Times* were told how in the hillside village of Cefn in northeast Wales ‘the long-continued drought [in 1896], and the consequent scarcity of water, make our gutters and our drains anything but pleasant. The stench is very obnoxious, and must be an important factor in spreading the fever’.^85^ Such was the alarm that any outbreak of disease during the ‘Long Drought’ came to be associated with drinking polluted water. Speaking about the area around the parish of Llandwrog in northwest Wales during the 1893 drought, one vestryman told the story of ‘a number of children returning home from school’ who ‘went a considerable distance in search of water to quench their thirst’. The result was that ‘three of them were now suffering from scarlet fever as a result of drinking water from a polluted well’.^86^

Fears about drought and disease were partly a response to alarm that existing waste removal practices in market towns and larger villages, such as the flushing of drains, to prevent outbreaks of disease had to be suspended during periods of water scarcity.^87^ At one level this highlights sanitary officials’ on-going faith in engineering solutions to prevent disease, the power of traditional associations between bad smells and disease, and just how fragile the sanitary state of many rural communities was perceived to be. However, with rural waste removal practices often basic beyond larger villages, fears about how drought led to outbreaks of disease were mainly articulated in the context of where rural communities were getting their water from as local supplies dried up. During the 1874 drought, the LGB noted that ‘there is reason to be apprehensive’ about the ‘danger to health, which will arise if, for want of a better supply, recourse is had to polluted water’.^88^ By the 1890s, such fears had become widespread in rural Wales. As the *Western Mail* worried, ‘with the usual sources of water supply already dried up in rural districts, there will naturally be a great temptation to farmers and cottages to resort to disused wells, or, for certain domestic purposes, to what is left of even usually stagnant pools’. For the newspaper, the consequences of this were ‘disastrous’.^89^ Such comments in the Welsh press contained within them both a clear sense of the link between polluted water and disease and assumptions of rural backwardness, but the resort to marginal sources of water was also framed as transgressive in its rejection of sanitary advice as sanitarians branded those using such water as irresponsible.

It would be easy to succumb to a narrative that blamed rural inhabitants’ use of polluted water supplies on their ignorance or lack of hygiene. However, rather than being passive, those living in rural Wales often had little option but to turn to marginal, disputed and polluted sources of water in times of drought as ‘every available source of water [was] utilised to obtain water for drinking’.^90^ Such responses could be framed as a rejection of advice from sanitary officials in favour of local knowledge and tradition; a rejection that hints at the limits of sanitary authority. However, as already suggested, the inhabitants of rural communities were conscious of the dangers of polluted water supplies. Evidence from rural sanitary officials and the press suggests that those living in rural communities appeared aware that ‘where people are obliged to drink water which must be polluted’ it was ‘likely to cause all sorts of disease’.^91^ Drought overrode these concerns.

Notwithstanding reminders that ‘all dwellers in rural districts’ should ‘under no stress of circumstances … resort to unwanted water supplies until they have been assured by some competent sanitary or medical advisor that they may be used with safety’, few of those facing water shortages followed this advice during drought conditions.^92^ With a different perception of the risk produced by the failure of local water supplies deemed safe, those living in villages and hamlets reacted under stress and turned to problematic or polluted supplies. At one meeting on rural water supplies during the 1887 drought, one speaker explained how people ‘were resorting to all the old wells they could find, and were opening up many which did not yield a pure supply’.^93^ For example, during the 1887 drought, the inhabitants of the large village of Pentyrch in south Wales were ‘reduc[ed] to using a disused well’ in which the local pigs wallowed. Others found that the wells they were using had dead dogs and cats at the bottom or, as the clerk of the Holywell Rural Sanitary Authority told the LGB, had ‘dead toads and vermin floating on the surface’, but continued to use the water from them for want of alternatives.^94^ Yet others turned directly to rivers and streams they knew to be polluted. Vestry officials in Peterston-super-Ely in the Vale of Glamorgan wrote to the Cardiff Union Rural Sanitary Authority in 1891 expressing alarm about how ‘when the rainfall is small inhabitants had to rely on the River Ely for drinking water’, which they knew was heavily polluted from tinplate works and collieries upstream. Members of the Llanelli Rural District Council were informed how, in response to record low rainfalls, tenants and farmer labourers already struggling with the effects of a depression in trade ‘are actually compelled to use ditch water for drinking purposes’ in the more isolated communities in the district.^95^ During periods of scarcity, overlapping anxieties about rural water supplies did not focus on tensions between consumers and water companies over practices and expectations, but on disease and access to drinking water. While it is important not to underestimate the role of public health concerns in shaping debates about water scarcity in urban areas, given the need for rural inhabitants to resort to marginal or polluted water supplies during drought, rural officials above all feared outbreaks of those diseases most associated with polluted water.

## Drought and Sanitary Improvements

It was against this background of mounting concern about access to water and fears about disease that rural sanitary authorities responded to drought. Following the 1872 Public Health Act, sanitary legislation was applied to rural areas and rural sanitary authorities were established under the control of existing poor law Boards of Guardians. Although they were excluded from administering certain sections of the 1872 and 1875 public health acts related to scavenging, street cleansing, highways or slaughterhouses—urban powers had to be sought on a case-by-case basis—it was the new rural sanitary authorities that were faced with the task overseeing sanitary reform for districts that had seen very little sanitary work.^96^ Under the 1878 Public Health (Water) Act, rural sanitary authorities were further required to provide ‘satisfactory supplies of water for all occupied dwellings’. These newly formed rural sanitary authorities quickly found themselves confronted with the problem of water scarcity. Some initially adopted temporary solutions to meet basic water needs: Wrexham Rural Sanitary Authority, for instance, promptly followed the LGB’s advice in 1874 and made ‘every precaution for the storage of wholesome water’ in the district, while in the Swansea rural district, water carts were used in 1887 to bring water to hard to reach villages.^97^ However, as drought became a more common occurrence after 1885, an analysis of newspaper reports on sanitary activity and of MOH reports and correspondence from forty-nine rural sanitary authorities reveals how across Wales rural authorities put in place schemes to improve rural water supplies as existing supplies came under intense scrutiny.^98^

In response to water scarcity, rural MOHs increasingly pressed for improved supplies and, just like their urban counterparts, cited the threat of cholera, typhoid and other waterborne diseases in their demands. For example, ratepayers in the market town of Llangollen in northeast Wales were stridently told in 1892 that their water supply was only likely to last four days in drought conditions and that unless they were ‘content to jog on with the solemn possibility of fever overtaking the community at any moment’ £6,700 had to be spent on an improved water supply.^99^ William Williams, Glamorgan’s county medical officer, warned that should the ‘great scarcity’ of 1893 be repeated there would be ‘an absolute water famine in the near future if some means are not taken to provide an adequate supply’.^100^ If improved rainfall in 1894 served temporarily to relieve ‘the scarcity of water’ in many rural districts, as the MOH for the Abergavenny Rural Sanitary District hoped in his 1894 report, ‘it will not be forgotten how serious matters may become in a year of drought’.^101^ Other rural medical officers in north Wales echoed these sentiments and pressed sanitary authorities to ensure that all rural communities had access to adequate water supplies.^102^

The anxieties voiced by rural sanitary officials were matched by local calls for better supplies. Although in rural Wales there was no collective consumer demand on the scale generated by the 1890s water shortages in East London, from the late 1880s parishes and villages responded to drought by calling on rural sanitary authorities to improve water supplies.^103^ These pleas reflected the growing agency of rural parishes and individuals in seeking action from rural sanitary authorities. The isolation and small size of many communities meant that any protest against poor or polluted supplies was often limited and localised. Yet, notwithstanding these limitations, central to the appeals made by rural communities for better supplies were experiences of water scarcity and concerns about disease, not demands for piped or constant supplies shaped by consumer politics or a hygienic gospel.

While there was considerable topographical, social and political diversity between rural sanitary authorities in Wales, in the face of water scarcity, fears about disease, pressure from MOHs and demands from communities, drought acted as a catalyst for rural sanitary intervention and improving local water supplies. With private communities playing a marginal role in suppling rural communities in the Principality, rather than political solutions and a municipalisation of existing supplies being suggested, as Trentmann shows for urban water consumers during ‘the Long Drought’ or Eccleston for North Yorkshire, rural sanitary authorities in Wales focused on local, practical solutions.^104^ As Elmes Steele, MOH for the Abergavenny Rural District Council explained in 1903, with rural districts ‘always liable to a period of drought …  we ought to prepare for it when it comes’.^105^ For example, in the hillside village of Craig Trebanos, the provision of a water tank by the Pontardawe Rural Sanitary Authority following the 1893 drought as part of the authority’s energetic ‘attention paid to defective w. supplies’ was declared an ‘important improvement’ and of ‘great satisfaction’ to local inhabitants.^106^ In 1898 the Holywell Rural District Council reported ‘distinct improvements’ in its water supplies after two years of work. Even though in other areas of sanitation the LGB found ‘little or no advance’ given disagreements within the council, by 1905 the most populous parts of the Holywell rural district had a piped supply.^107^ Encouraged by the LGB to take ‘acts necessary for providing a water supply for their districts’, those rural sanitary authorities bordering larger towns entered into arrangements with nearby urban authorities to extend water supplies to villages, although not without lengthy discussions about cost.^108^ A small number of rural authorities invested in larger water infrastructure projects. New local reservoir schemes were investigated, debated and, once the issues surrounding boundaries, responsibilities, and finances were resolved, built. In Henllan, a reservoir ‘sufficient for two months’ drought was eventually built, while in mid-Wales ‘dry weather’ drove the Rhayader Rural Sanitary Authority to enlarge its reservoir to meet the needs of the agricultural communities in the district.^109^ Speaking at the dinner to mark the opening of the reservoir at Llyn Dulyn in 1888, the chairman of the Llandudno Improvement Commission explained how the deepening of the lake placed the surrounding communities in a better position to cope with drought in the future.^110^

With many rural sanitary authorities worried about limited water supplies, protests were made against moves by English municipalities to build their reservoirs in Wales as the 1880s and 1890s saw large-scale schemes by Liverpool and Birmingham to capture rural Welsh watersheds.^111^ As Welsh rural water supplies were perceived to come under threat, the *Western Mail* warned that unless something was done, ‘the watersheds and lakes of Wales will have been “sucked dry” by English towns and cities’, forcing communities to fall back on ‘the dirty water of the mountain swamps’ as they had done during earlier droughts.^112^ In response to plans by the London County Council to use Welsh water to meet the capital’s growing needs, rural authorities in Glamorgan banded together in 1895 to protest against ‘the rapidly decreasing area in Wales available as gathering ground for a supply of water’ to ‘secure the future Water supply of the constituent districts’.^113^ The London scheme was abandoned in 1900 in the face of opposition, but throughout the Edwardian period, rural communities in other parts of Wales voiced apprehension about how large towns were gaining control of local springs, creating problems during droughts.^114^ Although the interests of urban areas took precedence, these protests reveal ongoing fears about the impact of drought and the need to secure better rural water supplies in the face of repeated periods of scarcity.

For many rural areas in Wales, reservoir building or extending piped water supplies were not feasible—economically or practically. Most plans to improve rural water supplies hence remained small scale, which accounts for why rural sanitary authorities in England and Wales borrowed a fifth of the amount loaned to urban authorities for waterworks between 1886 and 1897.^115^ Rather than being examples of modernisation or municipalisation—concepts which fail to adequately describe the work undertaken by rural sanitary authorities—the small-scale localised approaches adopted to improve rural water supplies reflected the limitations of rural sanitary reform. Low population densities and high outmigration ensured that rural communities lacked many of the preconditions—population growth, civic pride, party-political activity, commercial demand—favourable to sanitary reform.^116^ Rural poverty meant they had fewer financial resources to draw upon, while large parishes and scattered settlements could mean that other villages in the parish could resist improvements in one village.^117^ Rural officials were equally acutely aware that sanitary legislation was designed to deal with urban problems and that they lacked the same powers as their urban counterparts, but many rural communities in Wales were also isolated, hard to reach places, which made providing them with water both difficult and expensive. These problems were compounded by geology and mountainous terrain and the expense of getting water to remote communities. The combination of environment, landscape, and the isolated nature of many rural communities hindered rural authorities in developing the sanitary infrastructures found in even the smallest towns.

In thinly populated and often isolated rural districts, many Welsh sanitary authorities felt that networks of piped water were of little utility or value. Instead, local, low-cost solutions were considered the most appropriate, cost-effective responses so that ‘what has occurred in previous dry summers may not occur again’.^118^ For example, in the ‘rough, mountainous, and mainly agricultural’ Dolgellau Rural Sanitary Authority, the solution to local water scarcity caused by the 1893 drought was to pipe water to ‘a convenient spot’ at ‘a very small cost’ in an environment where the authority and MOH took ‘great trouble’ to tackle sanitary problems.^119^ MOH reports submitted to the LGB reveal how the main response to drought was for rural authorities to sink new wells or improve existing wells or springs, solutions that often took months rather than involving rural sanitary authorities in heated or protracted debates.^120^ For instance, in the ‘almost entirely agricultural’ district covered by the Cowbridge Rural District Council in south Wales, work to improve water supplies dominated the months after the 1895 and 1896 droughts and concentrated on repairing and deepening existing wells.^121^ The poorer Valley Rural District Council on the west coast of Anglesey responded in the same way to water scarcity. Following the severe drought of 1895, the council’s MOH reported ‘there is scarcely a hamlet or parish in the country where the necessity for a new well or pump has not been mooted’, while the conscientious Haverfordwest Rural District Council met the ‘very scanty’ supply of water ‘during long drought’ by sinking new wells for the scattered farming communities that made up the district.^122^ New wells or improving existing supplies were not just short-term solutions or measures adopted because they tended to be uncontentious. They reflected perceived local needs, the nature of local water supplies, topography, the isolation of many rural communities, and the resources available. As the MOH for Chirbury Rural District Council on the Welsh–English border commented in 1898, ‘Strongly as I disapprove … . of the sinking of additional wells, I fear I can offer no better suggestion than the sinking of two fresh wells in the most suitable parts of the village’ of Worthen to improve the water supply.^123^ It was not until the 1920s that many rural sanitary authorities were in a position to undertake more ambitious schemes.

## Conclusion

By the Edwardian period, Welsh rural sanitary authorities had become more water conscious and although complaints seldom drew on a consumerist language found in urban areas, rural communities had become quicker to call on rural district councils to improve supplies to address problems of water scarcity. Considerable attention was paid to defective and inadequate rural water supplies, even if the work of rural sanitary authorities favoured small-scale solutions rather than a municipalisation of existing supplies and a shift to piped networks of water found in urban communities. Although problems remained, especially in ‘outlying’ rural parishes where water scarcity remained common during the summer, drought drove improvements to local water supplies.^124^ By 1901, newspapers were reporting how drought, if still a problem for rural communities, was causing less ‘immediate anxiety than on previous occasions …  owing to more Extended provision of water supply and storage’.^125^

This is not to suggest that efforts to extend access to clean water went uncontested. Although many local, small-scale measures to improve supplies seemingly generated little debate, once the immediate drought conditions eased, schemes considered too costly were resisted and local sources of supply were defended from shared use as part of what Reay has described as ‘the background noise of nineteenth-century protest’ in rural communities.^126^ Questions of local finance, land ownership and lengthy debates about the merits of different solutions could dog more extensive improvements, ensuring that ambitious or expensive schemes were shelved or delayed, such as in Caernarvonshire where the county MOH lamented that the ‘owners of lakes and land place obstacles in the way of reform’.^127^ Although rural sanitary authorities tended to side with tenants against landholders when it came to improving water supplies, rural MOHs expressed frustration that more was not being achieved beyond the sinking of new wells or improving existing supplies, hinting at the tensions that could exist within local boards or between rural authorities and parishes.^128^ This exasperation is evident in the outburst from Llangyfelach Rural Sanitary Authority’s MOH, E. Rose Morgan, when he asked

How many years have I been crying out for an extended supply for Llansamlet? I am well-nigh sick and tired of preaching from the same text to such a deaf audience as your Board. Every summer all the inhabitants cry out; the winter comes and there is a full tap, and thus the years go and nothing is done!^129^

However, as Elmes Steele, MOH for Abergavenny Rural Sanitary District, commented in 1898, though medical officers would much rather have seen a more comprehensive water scheme introduced than those implemented, ‘we, I suppose, must be thankful for small mercies’ in times of drought.^130^

An examination of rural communities rather than towns reveals the considerable problems villages and rural parishes faced in times of drought, problems that were highly visible in Wales but also reported in English rural communities.^131^ What emerges is how experiences of drought were shaped as much by meteorology and hydrology as they were by the fragility of rural water supplies. In rural communities these produced substantial vulnerabilities to periods of water scarcity. Drought brought into sharp contrast the precarious nature of rural supplies, creating hardships that markedly impinged on the patterns of everyday life. Notwithstanding local knowledge that some supplies were dangerous because they were polluted, droughts forced those living in rural communities to turn to marginal and polluted sources of water.

Reports in the Welsh press about how rural inhabitants were exposed to an increased risk of disease reveal the importance of the connections made between drought and disease, but they also demonstrate how drinking and water-use practices were modified in the face of climatic events and how considering such events draws attention to the relationship between weather and public health. In turn, the responses to drought by rural sanitary authorities highlight the role of environmental stressors in public health. Fears about drought and disease, rather than about ideas of cleanliness or consumer politics, created an important stimulus for rural sanitary authorities to improve water supplies to resolve the tensions drought revealed between material, environmental and human agencies. Whereas urban public health concerns surrounding drought were linked to the making of a modern consumer, in a rural context, drought raised crucial questions about access to water, not constant (piped) supplies, cost or the responsibilities of private companies. While the approaches to improving rural supplies examined here shows how the work of rural sanitary authorities needs to be integrated into public health histories, this essay also exposes how rural supplies do not conform to existing accounts of water provision. In doing so, it suggests how ideas about municipalisation and the importance of networks of piped water need to be modified for different environments and communities.

## Footnotes

1 ‘Government Inquiry at Denbigh’, *Wrexham Advertiser, and North Wales News*, 3 May 1890, 3.

2
*Wrexham Advertiser, and North Wales News*, 14 May 1892, 8.

3 Met Office Hadley Centre Observation Data, Parallel Series of HadUKP data, <http://www.metoffice.gov.uk/hadobs/hadukp/data/simdownload.html>, accessed 16 July 2016.

4
*Caernarvonshire Combined Sanitary Districts Report for the Year ending December 31^st^ 1895* (Caernarvon, 1896), 29.

5 Alison Bashford and Sarah W. Tracy, ‘Introduction: Modern Airs, Waters, and Places’, *Bulletin of the History of Medicine*, 2012, 86, 495–514, 495.

6 See Bill Luckin, *Pollution and Control: A Social History of the Thames in the Nineteenth Century* (Bristol and Boston: Adam Hilger, 1986); Christopher Hamlin, *A Science of Impurity: Water Analysis in Nineteenth Century Britain* (Berkeley: University of California Press, 1990); John Hassan and Peter Taylor, *The Politics of Water in Early and Mid-Victorian Britain* (Manchester: Manchester University Press, 1996); Christopher Hamlin, *Public Health and Social Justice in the Age of Chadwick* (Cambridge: Cambridge University Press, 1998); John Hassan, *A History of Water in Modern England and Wales* (Manchester: Manchester University Press, 1998); Stephen Halliday, *The Great Stink: Sir Joseph Bazelgette and the Cleansing of the Victorian Metropolis* (Stroud: The History Press, 1999); Patrick Joyce, *Rule of Freedom: Liberalism and the Modern City* (London: Verso, 2003), 66–70.

7 On approaches to natural disasters, see Moncia Juneja and Franz Maulshagen, ‘Disasters and Pre-Industrial Societies: Historiographical Trends and Comparative Perspectives’, *Medieval History Journal*, 2007, 10, 1–31; Gerry Schenk, ‘Historical Disaster Research. State of Research, Concepts, Methods and Case Studies’, *Historical Social Research*, 2007, 32, 9–31. On drought, see for example, Richard G. Robbins, *Famine in Russia, 1891–1892* (New York: Columbia University Press, 1975); Donald Worster, *Dust Bowl: The Southern Plains in the 1930s* (Oxford: Oxford University Press, 1979); Mike Davis, *Late Victorian Holocausts. El Niño Famines and the Making of the Third World* (London: Verso, 2011); Georgina Enfield, *Climate and Society in Colonial Mexico* (Malden, MA: Wiley-Blackwell, 2008). On historic patterns of drought in England and Wales, see H. J. Fowler and C. G. Kilsby, ‘A Weather-type Approach to Analysing Water Resource Drought in the Yorkshire Region from 1881 to 1998’, *Journal of Hydrology*, 2002, 262, 177–92; Vanessa Taylor *et al.*, ‘Drought is Normal: The Socio-technical Evolution of Drought and Water Demand in England and Wales, 1893–2006’, *Journal of Historical Geography*, 2009, 35, 568–91; Muhammad Rahiz and Mark New, ‘Spatial Coherence of Metrological Droughts in the UK since 1914’, *Area*, 2012, 44, 400–10.

8 Ian Douglas, *Cities: An Environmental History* (London: I.B. Tauris, 2013), 211–51.

9 See Phil D. Jones *et al*., eds, *History and Climate: Memories of the Future?* (London: Springer, 2001).

10 Taylor *et al*., ‘Drought is Normal’, 568–91; Vanessa Taylor and Frank Trentmann, ‘Liquid Politics: Water and the Politics of Everyday Life in the Modern City’, *Past and Present*, 2011, 211, 199–241; Frank Trentmann and Vanessa Taylor, ‘From Users to Consumers: Waters Politics in Nineteenth-century London’, in Frank Trentmann, ed., *The Making of the Consumer: Knowledge, Power and Identity in the Modern World* (Oxford: Oxford University Press, 2006), 53–79.

11 ‘Country Notes’, *Country Life Illustrated*, 24 June 1899, 771.

12 For an overview of the literature on rural public health in Britain and Europe, see Astri Andresen, Josep L. Barona and Steven Cherry, ‘Introduction: “Rural Health” as a European Historical Issue’, in Astri Andresen, Josep L. Barona and Steven Cherry, eds, *Making a New Countryside: Health Policies and Practices in European History c.1860–1950* (Frankfurt am Main: Peter Lang, 2010), 11–24.

13 Existing scholarship on Wales has examined the politics of urban water supplies and quasi-imperialist efforts to bringing clean water to English cities, but little is known about water provision in the Principality: John Wyn Pritchard, ‘Water Supply in Welsh Towns, 1840–1900: Control, Conflict and Development’, *Welsh History Review*, 2002, 21, 24–47; Owen R. Roberts, ‘Developing the Untapped Wealth of Britain’s “Celtic Fringe”: Water, Engineering and the Welsh Landscape, 1870–1960’, *Landscape Research*, 2006, 31, 121–33; Owen R. Roberts, ‘Waterworks and Commemoration: Purity, Rurality, and Civic Identity in Britain, 1880–192’′, *Continuity and* Change, 2007, 22, 305–25; Richard Coopey, ‘Politics, Imperialism and Engineering: The Construction of the Birmingham Welsh Water System, 1861–1952’, in J. A. A. Jones, ed., *Water in the Celtic World: Managing Resources for the 21st century : 2nd Inter-Celtic Colloquium, University of Wales, Aberystwyth, 3–7 July 2000* (Wallingford: Centre for Ecology and Hydrology on behalf of the British Hydrological Society, 2000), 375–8.

14 On the concept of social vulnerability, see Anthony Oliver-Smith, ‘Theorizing Vulnerability in a Globalized World’, in Greg Bankoff, Georg Frerks and Dorothea Hilhorst, eds, *Mapping Vulnerability: Diseases, Development and People* (Sterling, VA: Earthscan, 2004), 10–24.

15 See Barry Reay, *Microhistories: Demography, Society and Culture in Rural England 1800–1930* (Cambridge: Cambridge University Press, 1996).

16 For a survey of these ideas, see Jeremy Burchardt, *Paradise Lost: Rural Idyll and Social Change since 1800* (New York: I.B. Tauris, 2002), 95–100.

17 Taylor and Trentmann, ‘Liquid Politics’, 221.

18 Frank Trentmann, *Empire of Things* (London: Allen Lane, 2016), 178–90.

19 See Christopher Hamlin, ‘Muddling in Bumbledom: On the Enormity of Large Sanitary Improvements in Four British Towns, 1855–1885’, *Victorian Studies*, 1988, 32, 55–83; Anthony S. Wohl, *Endangered Lives: Public Health in Victorian Britain* (London: Methuen, 1984), 167; Anne Digby, ‘The Local State’, in E. J. T. Collins, ed., *The Agrarian History of England and Wales: Volume VII 1850–1914* part 2 (Cambridge: Cambridge University Press, 2000), 1425–64; F. M. L. Thompson, ‘Landowners and Rural Community’, in G. E. Mingay, ed., *The Victorian Countryside*, 2 vols (London: Routledge, 1981), II, 457–74.

20
*Water Undertakings (England and Wales)*, PP (1914) 395, xxxvii.

21 Merthyr Tydfil Rural Sanitary District Annual Report, 1874, GA, D404/1/1.

22 ‘Sanitation in Rural Districts’, *Weekly News and Victors’ Chronicle for Colwyn Bay*, 30 March 1900, 7.

23
*Dr. W. W. E. Fletcher’s Report to the Local Government Board upon the Sanitary Circumstances and Administration of the Aberayron Registration District, comprising the Rural District of Aberayron and the Urban Districts of Aberayron and New Quay in Cardiganshire* (London: Darling & Son, 1907).

24 Lithiby to Abergavenny Rural District Council (RDC), 21 November 1907, MH 97/118, The National Archives (hereinafter TNA).

25 William Williams, *Glamorgan County Council Annual Report of the County Medical Officer for the Year 1893* (Cardiff: William Lewis, 1894), 70; Petition sent by the inhabitants of Cilycwm village to the Sanitary Board at Llandovery, 6 July 1893, MH 12/15948, TNA.

26 Cardiff Union Rural Sanitary Authority (RSA), MOH annual report, 1875, D805/4/1, 1886, D805/4/1, GA.

27 Hawarden RSA, MOH annual report, 1880, MH 97/140, TNA; William Williams, *Glamorgan County Council Annual Report of the County Medical Officer for the Year 1896* (Cardiff, 1897), 75; Gower RSA, MOH annual report, 1888, MH 95/136/.

28
*Dr. W. W. E. Fletcher’s Report to the Local Government Board*, 3–4; *Mr. Spear’s Report to the Local Government Board on an Outbreak of Diphtheria in the Dingestow Registration sub-district of the Monmouth Rural Sanitary District* (London: Eyre and Spottiswoode, 1888); Llanfyrnach RDC, MOH annual report, 1896, MH 97/149, TNA; Chirk RDC, MOH annual report, 1902, MH 97/125, TNA.

29 A Summary of the Annual Reports of the Medical Officers of Health of Several Sanitary Districts in the Administrative County of Cardigan for 1906, MH 97/4, TNA.

30 William Williams, *Annual Report of the County Medical Officer for the Year 1901* (Cardiff: William Lewis, 1902), 30–2.

31 See Hamlin, *A Science of Impurity*.

32 Cardiff Union RSA, Report of the Surveyor and Nuisance Inspector, 1874, UC/91/2, GA; MOH annual report, Dolgellau district, 1890, MH 97/130, TNA.

33 Williams, *Glamorgan County Council Annual Report of the County Medical Officer for the Year 1894* (Cardiff: William Lewis, 1895), 105.

34 Merthyr Rural Sanitary District, annual report, 1881, D404/1/8, GA; Bridgend and Cowbridge RSA minutes, 3 January 1874, UB/68/1, GA; Letter to the Chairman of Aberaeron RDC, 8 February 1896, Provincial MOH reports, Wellcome Library, London.

35 ‘Sanitation in Glamorgan’, *Western Mail*, 8 September 1894; T. Evans, ‘A Plea for Welsh Streams’, *Fishing Gazette*, 10 November 1894, 460; W. Fraser, *River Pollution Report*, 13 August 1890, Cardiff Union RSA, GC/JR/2/1, GA.

36 Annual Report of the MOH for the Rural Sanitary District of Llandilo, 1894, MH 12//15936, TNA.

37
*Western Mail*, 8 July 1887; Cardiff Union RSA, annual report, 1876, D805/4/1, GA; Bridgend and Cowbridge RSA minutes, 17 May 1884, UB/68/3, GA.

38 Bridgend and Cowbridge RSA minutes, 18 December 1880, UB/68/2, GA; William Williams, *Glamorgan County Council Annual Report of the County Medical Officer for the Year 1897* (Cardiff: William Lewis, 1898), 33.

39 G. A. Cole and T. J. Marsh, *The Impact of Climate Change on Severe Drought: Major Droughts in England and Wales from 1800 and Evidence of Impact* (Bristol: Environment Agency, 2006); Crickhowell RDC, MOH annual report, 1911, MH 97/128, TNA; Anglesey County Council, MOH report, 1912, MH 97/1, TNA.

40 T. Marsh, G. Cole and R. Wilby, ‘Major Droughts in England and Wales’, *Weather*, 2007, 62, 87–93.

41 Taylor *et al*., ‘Drought is Normal’, 571.

42 ‘Water Supply of Rural Essex’, *British Medical Journal*, 1891, 1577, 674.

43
*Eighteenth Annual Report of the Local Government Board, 1888–89. Supplement Containing the Report of the Medical Officer for 1888* (London: HMSO, 1889), 213.

44 C. E. P. Brooks and J. Glasspoole, *British Floods and Droughts* (London: Ernest Benn, 1928); 135–36.

45 Met Office Hadley Centre Observation Data, Parallel Series of HadUKP data, <http://www.metoffice.gov.uk/hadobs/hadukp/data/simdownload.html>, accessed 16 July 2016.

46 Trevor A. Harley, ‘Nice Weather for the Time of Year: The British Obsession with the Weather’, in Sarah Strauss and Benjamin S. Orlove, eds, *Weather, Climate and Culture* (Oxford: Berg, 2003), 103–18.

47
*Western Mail*, 8 July 1887.

48
*‘*The Excessive Drought’, *Wrexham and Denbighshire Advertiser*, 2 July 1887, 7; ‘The Drought at Swansea’, *Western Mail*, 28 June 1887; ‘Short Supply at Cadoxton’, *Western Mail*, 1 July 1887.

49 R. C. Mossman, ‘The Dry Summer of 1913’, *British Rainfall, 1913. On the distribution of rain in space and time over the British Isles during the year 1913 as recorded by about 5,500 observers in Great Britain and Ireland and discussed with articles upon various branches of rainfall work* (London: Edward Stoppard, 1914), 35–4; ‘A Year of Drought’, *County Observer*, 27 August 1904, 7; Chepstow RDC, MOH annual report, 1900, MH 97/124, TNA.

50 J. Brodie, ‘The Great Drought of 1893’, *Quarterly Journal of the Royal Meteorological Society*, 1894, 20, 1–30; Williams, *Glamorgan County Council Annual Report of the County Medical Officer for the Year 1893*, 24.

51 ‘The Drought in South Wales’, *Western Mail*, 28 April 1893.

52 ‘The Drought’, *Western Mail*, 5 May 1893; ‘The Great Drought’, *Aberdare Times*, 24 June 1893.

53 ‘What the Drought means’, *Western Mail*, 11 May 1893.

54 ‘Water Famine in the Vale of Clwyd’, *Wrexham Advertiser*, 17 June 1893, 2; Shepherd to Hay RSA, 28 February 1894, MH 12/15782, TNA; *North Wales Express*, 14 July 1893, 2.

55 ‘Break-up of the Drought’, *Western Mail*, 16 May 1893.

56 T. W. Fletcher, ‘The Great Depression of English Agriculture, 1873–1986’, *Economic History*, 1961, 13, 417–32, 427; ‘Effects of the Drought’, *Evening Express*, 18 May 1893, 2; *Wrexham Advertiser*, 17 June 1893, 7; *First Report of the Royal Commission on Land in Wales and Monmouthshire* (London: HMSO, 1984), 140; ‘Farmers and the Drought’, *Cambrian News and Merionethshire Standard*, 23 June 1893, 5.

57 ‘Wales by Day’, *Western Mail*, 24 November 1893.

58 Met Office Hadley Centre Observation Data, Parallel Series of HadUKP data, <http://www.metoffice.gov.uk/hadobs/hadukp/data/simdownload.html>, accessed 16 July 2016.

59 ‘A Word of Warning’, *Evening Express*, 13 June 1898, 2.

60
*Twenty-seventh Annual Report of the Local Government Board, 1897–98. Supplement Containing the Report of the Medical Officer for 1897–98* (London: HMSO, 1998), xvii; Thomas to LGB, 22 February 1895, MH 12**/**15733, TNA; ‘Llysfaen Water Supply’, *Welsh Coast Pioneer*, 10 August 1900, 10.

61 Letter to the Chairman of the Aberaeron RDC, 8 February 1896, Provincial MOH reports, Wellcome Library; *Twenty-Sixth Annual Report on the Sanitary Condition of the Abergavenny Rural Sanitary District* (Abergavenny, 1898), 5; *Caernarvonshire combined Sanitary Districts Report for the Year Ending December 31^st^ 1893* (Caernarvon, 1894), 17; William Williams, *A Sanitary Survey of Glamorganshire* (Cardiff: Daniel Owen & Co, 1895), 9.

62 ‘What the Drought means’, *Western Mail*, 11 May 1893; ‘Llanfihangel-Geneu’r-Glyn’, *Welsh Gazette**and West Wales Advertiser*, 30 May 1901, 5; ‘Feared Water Famine’, *Weekly Mail*, 9 November 1901, 27; ‘Welcome Rain’, *Cheshire Observer*, 6 October 1904, 10; ‘Rhyl Water Supply’, *Rhyl Journal*, 22 September 1906, 5; ‘Cardiff without Water’, *Weekly Mail*, 13 June 1908, 4; ‘Burry Port’s Famine’, *Cambrian*, 13 August 1909, 8.

63 In 1910, the LGB collected information on urban and rural areas, producing composite reports on the nature of sanitation: see LGB sanitary papers, MH 12, TNA.

64 See, for example, ‘The Drought’, *Western Mail*, 29 June 1887; ‘Retrospect of the Year 1887’, *Wrexham Advertiser*, 31 December 1887, 7; ‘The Recent Drought’, *Cardiff Times*, 17 September 1887; *North Wales Chronicle*, 17 June 1893; Williams to LGB, 20 September 1893, MH 12/16521, TNA; Report for the Year 1893 on the Forden Rural Sanitary District, MH 12/16596, TNA; *Western Mail*, 22 June 1893; MOH annual report, St David’s District, Haverfordwest RSA, 1894, MH 12/16651, TNA; Williams, *Sanitary Survey of Glamorganshire*, 80; ‘Famine and Epidemic’, *South Wales Daily Post*, 25 August 1899; *Welsh Gazette and West Wales Advertiser*, 30 May 1901, 5; Rhayader RDC, MOH annual report, 1904, MH 97/167, TNA; Llanelli RDC, Annual Report of the Medical Officer of Health for the Year 1907, MH 97/147, TNA; Crickhowel RDC, Medical Officer’s Annual Report, 1911, MH 97/128, TNA.

65 ‘Scarcity of Water in the Rhymney Valley’, *Cardiff Times*, 8 August 1885, 8.

66 See, Taylor *et al*, ‘Drought is Normal’, 584; ‘The Drought’, *Western Mail*, 7 July 1887; ‘Waste of Water’, *Wrexham Advertiser*, 16 July 1887, 5; ‘Drought in the Rhondda District’, *Cardiff Times*, 22 June 1895; ‘The Drought’, *Evening Express*, 31 May 1895, 3.

67 See, for example, *Wrexham Advertiser*, 4 April 1884, 5; ‘The Drought’, *Western Mail*, 7 July 1887; ‘The Water Supply’, *Wrexham Advertiser*, 20 August 1887, 8.

68 ‘What the Drought means’, *Western Mail*, 11 May 1893.

69 B. Short, ‘Rural Demography, 1850–1914’, in E. J. T. Collins, ed., *The Agrarian History of England and Wales: Volume VII 1850–1914* part 2 (Cambridge: Cambridge University Press, 2000), 1271; Reay, *Microhistories*, 68–96; Anne Hardy, *The Epidemic Streets: Infectious Diseases and the Rise of Preventive Medicine, 1856–1900* (Oxford: Oxford University Press, 1993), 90; Denbighshire County Council, Annual Report on the Health of Denbighshire for the Year 1911, MH 97/8, TNA; B. Seebohm Rowntree and Mary Kendall, *How the Labourer Lives* (London: Thomas Nelson, 1913).

70 Cardiff Union RSA, Report of the Surveyor and Nuisance Inspector, 1873/4, UC/91/2, GA; Lloyd to Dogelley RSA, 23 November 1893, MH 12/16521, TNA.

71 ‘What the Drought means’, *Western Mail*, 11 May 1893.

72 Mary Dobson, *Contours of Death and Disease in Early Modern England* (Cambridge: Cambridge University Press, 1997), 397–9, 404–6, 415–17, 442–5.

73 ‘Water Famine’, *Western Mail*, 29 June 1887.

74 ‘The Drought at Swansea’, *Western Mail*, 28 June 1887; ‘Short Supply at Cadoxton’, *Western Mail*, 1 July 1887.

75 Cited in Trentmann and Taylor, ‘From Users to Consumers’, 58.

76 See, Evans to Anglesey Union RSA, 1893, MH 12/15690, TNA; Hill to Crickhowell RSA, 21 March 1893, MH 12/15768, TNA.

77 Williams, *Sanitary Survey of Glamorganshire*, 80.

78 Haynes to LGB, 25 April 1893, MH 12/16474, TNA.

79
*South Wales Daily Post*, 27 April 1893.

80 For example, an epidemic outbreak of fever in Middlesbrough in 1887-8 was attributed to drought conditions, with the same drought felt to exacerbate the conditions that led to typhoid in the Teesside region: *Eighteenth Annual Report of the Local Government Board, 1888–89*, 235; *Twenty-first Annual Report of the Local Government Board, 1891–92. Supplement in Continuation of the Report of the Medical Officer for 1891. Enteric Fever in the Tees Valley* (London: Eyre and Spottiswoode, 1893).

81 ‘A Water Famine’, *Cardiff Times*, 19 August 1899, 4.

82 ‘Contaminated Water Causes Typhoid Fever’, *Evening Express*, 12 August 1896; ‘Swansea Rural Sanitary Authority’, *Cambrian*, 14 July 1893; *North Wales Chronicle*, 24 June 1893; ‘The Drought’, *South Wales Daily Post*, 25 August 1899, 4.

83 ‘The Condition of Affairs in the Pontypridd District’, *Western Mail*, 15 September 1893.

84 ‘Serious Drought in the District: Alarming Reports’, *North Wales Chronicle*, 17 June 1893; ‘The Water Question’, *Prestatyn Weekly*, 10 June 1905, 3.

85
*North Wales Chronicle*, 17 June 1893; ‘Cefn’, *Merthyr Times*, 13 August 1896, 6.

86 ‘The Want of Rain’, *Western Mail*, 14 June 1893; ‘Serious Drought in the District: Alarming Reports’, *North Wales Chronicle*, 17 June 1893.

87 For instance, in Llanfihangel-Geneu’r-Glyn, the scarcity of water during drought conditions meant that ‘the sanitation of the village is neglected’ ensuring that ‘the danger of an epidemic disease becomes very evident’: *Welsh Gazette and West Wales Advertiser*, 30 May 1901, 5.

88 LGB circular, Water Supply, 29 July 1874, UC/95/2, GA.

89
*Western Mail*, 22 June 1893.

90 ‘Rhymney Valley’, *Weekly Mail*, 20 August 1887, 6.

91 ‘Serious Drought in the District: Alarming Reports’, *North Wales Chronicle*, 17 June 1893; Merthyr Tydfil Rural Sanitary District, annual report, 1879, 13, D404/1/6, GA.

92
*Western Mail*, 22 June 1893.

93 ‘The Drought’, *Western Mail*, 1 July 1887.

94 ‘The Recent Drought’, *Cardiff Times*, 17 September 1887; Davies to LGB, 1 November 1893, MH 12/16207, TNA.

95 Cardiff Union RSA minutes, 11 March 1891, UC/75/1, GA; Llanelli RDC, Annual Report of the Medical Officer of Health for the Year 1907, MH 97/147, TNA.

96 W. Roberston and Charles Porter, *Sanitary Law and Practice* (London: Sanitary Publishing Co., 1909), 3; Thomas Whiteside Hime, *The Practical Guide to the Public Health Acts* (London: Bailliere, Tindall and Cox, 1901); Thomas Rees, *The Public Health Act from a Rural Inspector of Nuisances’ point of view* (London, 1884), 9–10.

97 ‘Wrexham Rural Sanitary Authority’, *Wrexham Advertiser*, 11 July 1874, 5; ‘The Drought at Swansea’, *Western Mail*, 28 June 1887; ‘Swansea Rural Sanitary Authority’, *South Wales Daily Post*, 21 June 1893, 3; ‘The Drought at Swansea’, *Western Mail*, 28 June 1887.

98 MOH reports for Welsh rural sanitary authorities, digests and correspondence with the LGB, including details of borrowing, are held at The National Archives in MH 97.

99
*Wrexham Advertiser*, 4 June 1892, 8.

100 Williams, *Annual Report of the County Medical Officer for the Year 1893*.

101
*Twenty Second Annual Report on the Sanitary Condition of the Abergavenny Rural Sanitary District* (Abergavenny, 1895), 5.

102
*Caernarvonshire combined Sanitary Districts Report for the Year Ending December 31^st^ 1893* (Caernarvonshire, 1894), 17.

103 See, for example, ‘Our Water Supply’, *Cardiff Times*, 3 August 1889; ‘Government Inquiry at Denbigh’, Wrexham Advertiser, 3 May 1890, 3; MOH annual report, Mold Division, 1894, MH 97/142, TNA; Bridgend and Cowbridge RSA minutes, 17 June 1894, UB/68/5, GA; ‘Water Supply in Carnarvon Union’, *North Wales Express*, 11 August 1893, 8; *Weekly News and Visitors’ Chronicle for Colwyn Bay*, 10 August 1900, 2.

104 Trentmann, *Empire of Things*, 185–86; Bernie Eccleston, *Pumps, Pipes and Purity: The Turbulent Social History of Providing the Public with Enough Safe Water in the Thirsk District and North Yorkshire from 1875* (York: York Publishing Services, 2012).

105 Annual Report on the Sanitary Condition of Abergavenny RDC, 1903, MH 97/118, TNA.

106 Pontardawe RSA (Western Division) sanitary report for 1894, MH 12/16436, TNA.

107 ‘Rural Water Supplies and the Recent Drought’, *Rhyl Journal*, 11 July 1897, 4; MOH annual report, Holywell division, 1897, MH 97/142, *ibid.*, 1899; Annual Report of Dr James Williams, Medical Officer of Health for the Holywell District, 1905, MH 97/142, TNA.

108 LGB Circular, Water Supply, 29 July 1874, UC/95/2, GA; Cowbridge RDC minute book, 1895–98, RDCOW/C/1/1, GA; Gelligaer and Rhigos RDC minutes, 26 January 1899, 6 May 1899, 25 May 1899, RDGR/C/1/1, GA.

109 MOH annual reports, Rhayader division, 1887, 1892, MH 97/167, TNA.

110 ‘The Llandudno Water Supply’, *North Wales Chronicle*, 23 June 1888.

111 See, Roberts, ‘Developing the Untapped Wealth of Britain’s “Celtic Fringe”’, 121–33; Roberts, ‘Waterworks and Commemoration’, 305–25; Coopey, ‘Politics, Imperialism and Engineering’, 375–8.

112 ‘A Question for the County’, *Western Mail*, 22 June 1895.

113 Bridgend and Cowbridge RSA minutes, 17 August 1895; UB/68/5, GA.

114 ‘London Welsh Water Bill’, *Western Mail*, 15 February 1900, 3; *Royal Commission on Canals and Waterways* (London: HMSO, 1907), II, 35; Annual Report on the Health of Denbighshire for the Year 1912, MH 97/8, TNA.

115
*Twenty-Seventh Annual Report of the Local Government Board, 1897–98* (London: HMSO, 1896), cv. For example, Machynlleth RSA, borrowed £80 in 1895 to improve the water supply for the Pennal parish: Works of Water Supply, Estimates and Details, February 1893, MH 12/16578, TNA.

116 Keir Waddington, ‘“It might not be a nuisance in a country cottage”: Sanitary Conditions and Images of Health in Victorian Rural Wales’, *Rural History*, 2012, 23, 185–204.

117 For example, according to the LGB this happened in Mardy in Abergavenny Rural District Council which made improving the water supply to the hamlet difficult: Lithiby to Abergavenny RDC, 8 December 1908, MH 97/118, TNA.

118 MOH annual report, Barmouth division, 1896, MH 97/130, TNA.

119 Dolgelley RDC, MOH annual Report, 1910, MH 97/130; MOH annual report, Mallwyd Division, 1893, MH 97/130, TNA.

120 See, Lloyd to Newcastle-in-Emlyn RSA, March 1894, MH 12/15963, TNA; MOH annual report, Milford Division, 1896, Gower RDC, MOH annual report, 1897, MH 95/136, TNA; Gelligaer and Rhigos RDC minutes, 28 September 1899, RDGR/C/1/1, GA; Llanfyrnach RDC, MOH annual report, 1901, MH 97/149, TNA.

121 Cowbridge RDC minutes, 1895–98, RDCOW/C/1/1, GA; LGB notes on Annual Report for 1910, MH 96/617, TNA.

122 Evans to Valley RDC, April 1896, MH MH 12/15706, TNA; Haverfordwest RDC, MOH annual report, 1893, 1896, MH 95/138, TNA.

123 ‘Chirbury Rural District Council’, *Montgomery County Times*, 26 February 1898.

124 ‘Carnarvon Rural Sanitary Authority’, *North Wales Chronicle*, 20 May 1893; ‘The Water Question’, *Prestatyn Weekly*, 10 June 1905, 3.

125 William Williams, *Annual Report of the County Medical Officer for the Year 1900* (Cardiff: William Lewis, 1901), 85, 87, 90; Cardiff Union RSA, annual report, 1897, D805/4/2, GA; ‘Trawsfynydd Water Scheme’, *Carnarvon and Denbigh Herald*, 22 June 1906, 4; ‘Water Supply in S. Wales’, *Cardiff Times*, 8 June 1901, 2.

126 Barry Reay, *The Last Rising of the Agricultural Labourers: Rural Life and Protest in Nineteenth Century England* (Oxford: Oxford University Press, 1990), 188. For example, those living near Penygraig’s spring resisted its use to supply water to local communities in 1901. They regarded the proposed use of the spring as an attempt to rob them of their water and ‘time after time destroyed the main and removed the tank’: Annual Report on the Sanitary Condition of Gelligaer and Rhigos RDC, 1901, RDGR/M/1/1, GA.

127 Caernarvonshire County Council, Annual Report of the County Medical Officer of Health, 1913, MH 97/7, TNA.

128 For example, in Bodfari in Flintshire, the MOH recorded how ‘nothing further done’ to improve the water supply because the ‘PC will not accept DC’s proposals’, while in St Dogmaels Rural District Council plans to improve the water supply at Penybryn had ‘fallen through on a/c of disagreements between DC and households’: St Asaph (Flint) RSA, MOH annual report, 1898, MH 97/170, TNA; St Dogmaels RDC, MOH annual report, 1908, MH 97/171, TNA.

129 William Williams, *Glamorgan County Council Annual Report of the County Medical Officer for the Year 1899* (Cardiff: William Lewis, 1900), 88.

130
*Twenty-Sixth Annual Report on the Sanitary Condition of the Abergavenny Rural Sanitary District* (Abergavenny, 1898), 5.

131 See, for example, *Annual Report of the Medical Officer of Health to the Local Government Board for 1907–08* (London: HMSO, 1909).

